# Construction of Supramolecular Polymers with Different Topologies by Orthogonal Self-Assembly of Cryptand–Paraquat Recognition and Metal Coordination

**DOI:** 10.3390/molecules26040952

**Published:** 2021-02-11

**Authors:** Kai Wang, Yuan-Guang Shao, Feng-Zhi Yan, Zibin Zhang, Shijun Li

**Affiliations:** College of Material, Chemistry and Chemical Engineering, Hangzhou Normal University, Hangzhou 311121, China; wangkai19940829@163.com (K.W.); 2018111009009@stu.hznu.edu.cn (Y.-G.S.); fengzhi_yan@stu.hznu.edu.cn (F.-Z.Y.)

**Keywords:** supramolecular polymers, cryptands, paraquat, metal-coordination, self-assembly, host–guest chemistry

## Abstract

Recently, metal-coordinated orthogonal self-assembly has been used as a feasible and efficient method in the construction of polymeric materials, which can also provide supramolecular self-assembly complexes with different topologies. Herein, a cryptand with a rigid pyridyl group on the third arm derived from BMP32C10 was synthesized. Through coordination-driven self-assembly with a bidentate organoplatinum(II) acceptor or tetradentate Pd(BF_4_)_2_•4CH_3_CN, a di-cryptand complex and tetra-cryptand complex were prepared, respectively. Subsequently, through the addition of a di-paraquat guest, linear and cross-linked supramolecular polymers were constructed through orthogonal self-assembly, respectively. By comparing their proton nuclear magnetic resonance (^1^H NMR) and diffusion-ordered spectroscopy (DOSY) spectra, it was found that the degrees of polymerization were dependent not only on the concentrations of the monomers but also on the topologies of the supramolecular polymers.

## 1. Introduction

Because of their stimuli-responsive properties due to dynamic reversible non-covalent interactions, supramolecular polymers have attracted the increasing attention of chemists [1,2,3,4,5,6,7]. Compared with traditional polymers, supramolecular polymers can easily change topology through the introduction of dynamic non-covalent interactions such as multiple hydrogen bonding [8,9,10,11,12,13], metal coordination [14,15,16,17,18,19], host–guest interactions [20,21,22,23,24], and aromatic stacking [25,26]. Moreover, the activation energy to break a non-covalent bond in supramolecular polymers is relatively low, which affords them with the capabilities of self-healing and stimuli-responsiveness to pH, temperature, anions or cations, light, gas, and so on [27,28,29]. Therefore, supramolecular polymers have been employed as unique components with excellent macroscopic properties in the fields of smart devices, optoelectronic materials, biomedical materials, and other functional materials [30,31,32,33,34].

Although supramolecular polymers with different chain topologies, for example, linear [35,36,37,38,39], cross-linked [40,41,42,43], star-type [44,45,46], and branched [47,48,49,50], have been extensively studied, it is still a significant challenge to design supramolecular polymers with controllable topologies, which might further change their physical properties, such as molecular weight, polydispersity, and viscosity. Thus far, many supramolecular polymers based on crown ethers have been prepared [51,52,53]. However, only a few supramolecular polymers made from crown ether-based cryptands have been reported. Compared to the corresponding crown ethers, cryptands are better hosts for paraquat derivatives due to the introduction of additional binding sites, which provide them not only better preorganization of complex conformations but also higher association constants. Normally, the association constants (*K*_a_) of cryptands to the same guests can be 10^3^−10^4^ times higher than those of their analogous crown ethers [54,55,56,57,58]. On the other hand, as one of the most common but important non-covalent interactions, metal coordination is often described as a strong, highly directional, and versatile supramolecular force, which can produce self-assemblies with various coordination geometries [59,60,61,62,63]. By changing the central metal, it is easy to adjust the coordination number and direction of the ligands, thus realizing topological control of the target self-assemblies.

For these two reasons, we report here the construction of linear and cross-linked supramolecular polymers by using the orthogonal self-assembly of cryptand-based host–guest recognition and metal coordination interactions. By changing the metal linkers (bidentate organoplatinum(II) acceptor **5** or quadridentate acceptor Pd(BF_4_)_2_•4CH_3_CN) in one of the monomers, supramolecular polymers with controllable topologies were conveniently prepared. It was proven that the degrees of supramolecular polymerization were not only dependent on the monomer concentrations but also on their topologies.

## 2. Results and Discussion

### 2.1. Design, Synthesis, and Characterization of Cryptand **4**

**Scheme 1:**
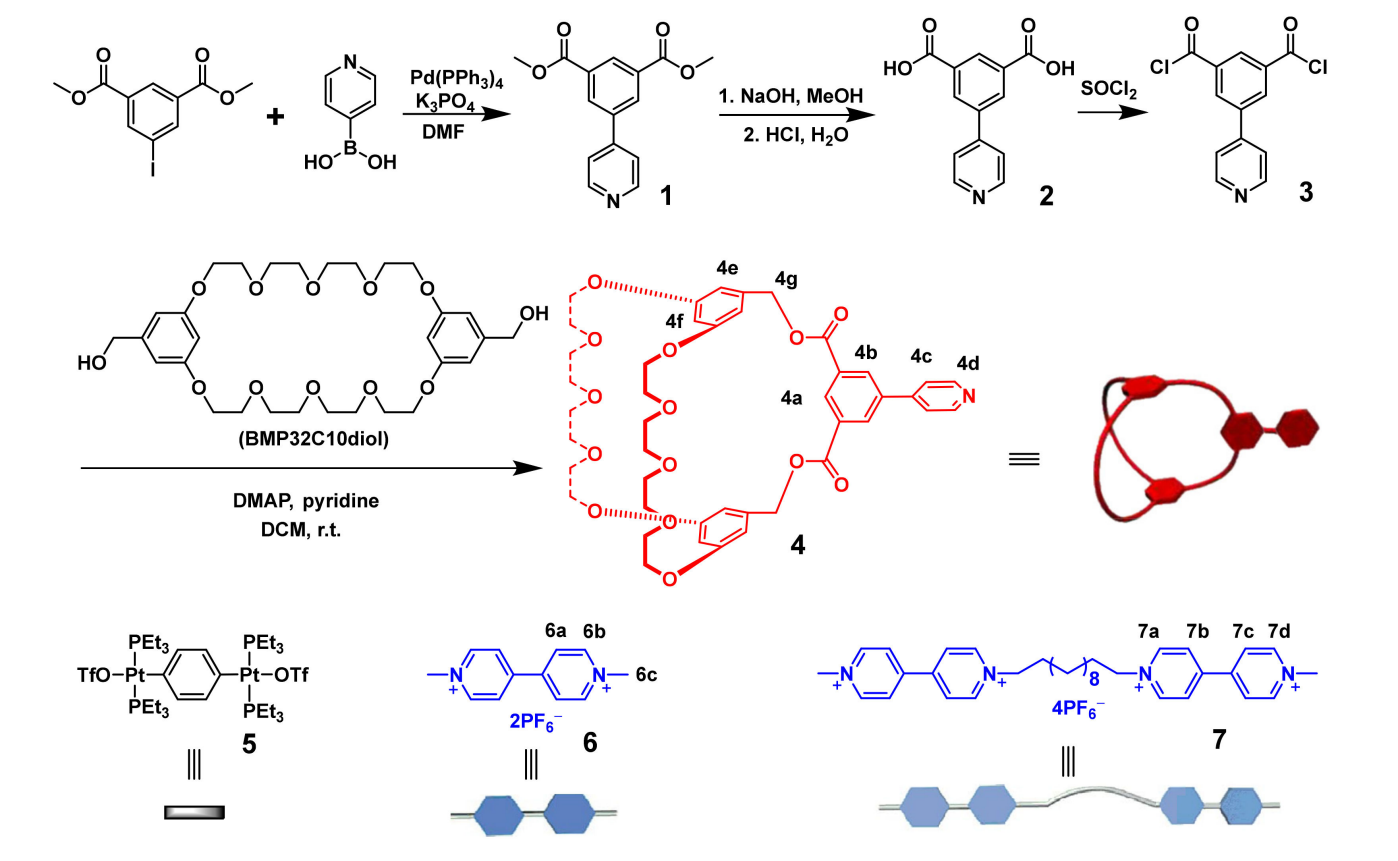
Synthetic routes to pyridyl-functionalized cryptand **4**; chemical structures and graphical representations of **4**–**7**.

To realize the orthogonal self-assembly and minimize the interference between the host–guest recognition and metal coordination, cryptand **4**, with a rigid pyridyl group on the third arm, was designed (Scheme 1). First, compound **1** was prepared by a Suzuki–Miyaura coupling reaction of dimethyl 4-iodoisophthalate and pyridine-4-ylboronic acid. After the hydrolysis and following acylchlorination, **3** and BMP32C10diol [64] were connected at a very low concentration in dichloromethane to produce cryptand **4** in 45% yield. The structure of **4** was clearly characterized by ^1^H and ^13^C{^1^H} NMR and electrospray ionization mass (ESI-MS) spectrometry (Figure S3, Figure S4 and Figure S5). The bidentate organoplatinum(II) acceptor **5** [65], paraquat **6** [66], and the di-paraquat guest **7** [67] were prepared following literature methods.

### 2.2. Investigation of the Host–Guest Complexation between Cryptand **4** and Paraquat

**Figure 1:**
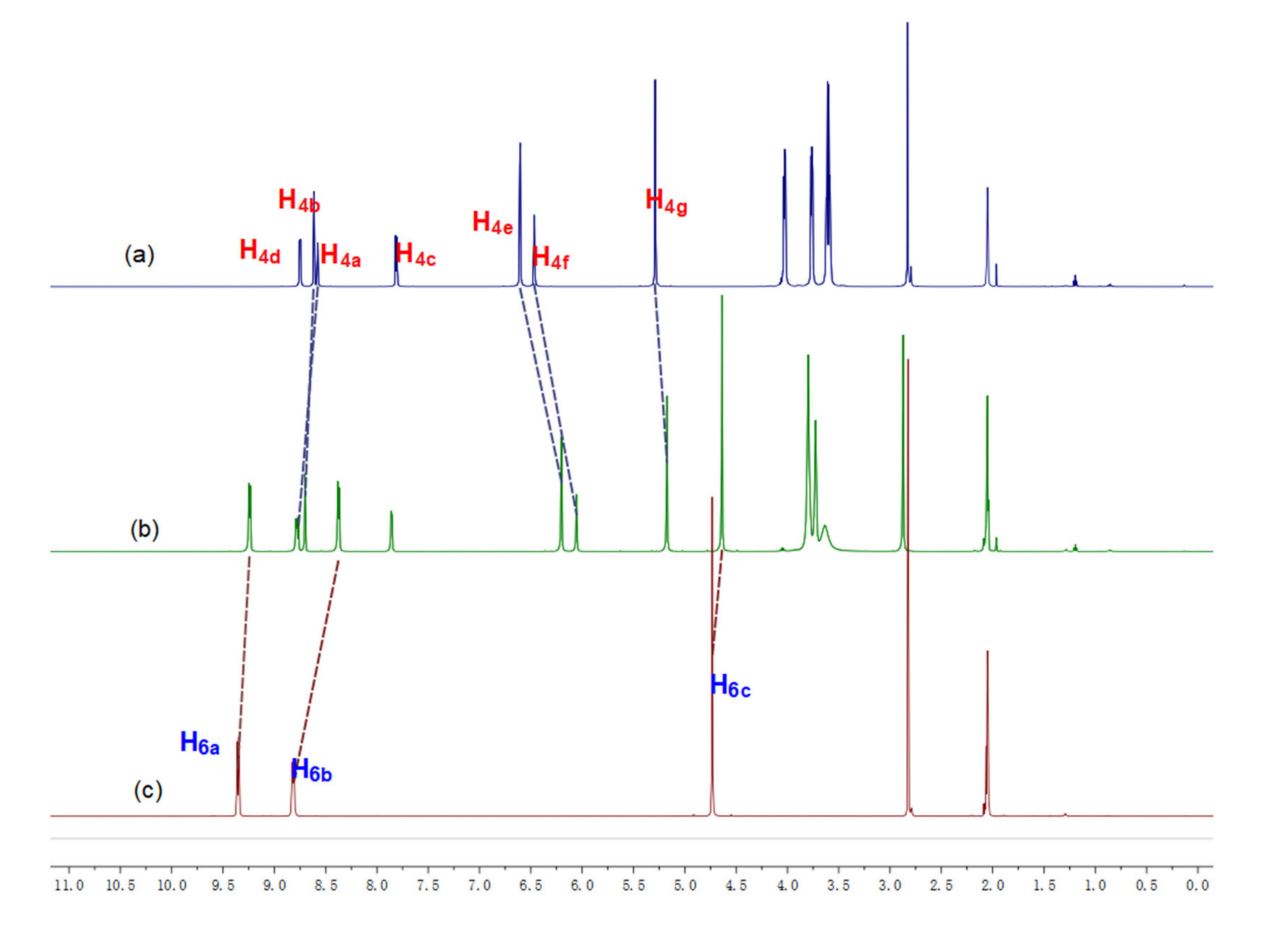
Proton nuclear magnetic resonance (^1^H NMR) spectra (500 MHz, CD_3_COCD_3_, 298K) of 5.00 mM **4** (**a**), 5.00 mM **4** + 5.00 mM **6** (**b**), 5.00 mM **6** (**c**). The assignments of protons were shown in Scheme 1.

To make sure that the third arm was located outside of the cryptand cavity, the host–guest recognition of **4** was investigated with paraquat **6** as a model guest. When a colorless 2.00 mM acetone solution of **4** and a colorless 2.00 mM solution of **6** were mixed, a yellow color resulted. The absorption spectrum of the mixture showed a new band (Figure S9) that can be attributed to the charge transfer between the electron-rich aromatic rings of **4** and the electron-poor pyridinium rings of **6**. A Job plot [68] (Figure S10) based on UV–vis absorbance data of the charge transfer band at *λ* = 400 nm demonstrated that their complex was of 1:1 stoichiometry in solution. The ^1^H NMR spectrum of a mixed solution of **4** (5.00 mM) and **6** (5.00 mM) in CD_3_COCD_3_ showed that the complexation is a fast exchange system (Figure 1b), just like other reported cryptand–paraquat systems [54,55,56,57,58]. The opposite chemical shift changes of their protons, including upfield shifts of H_6a_, H_6b_, H_6c_, H_4e_, H_4f_, and H_4__g_ and downfield shifts of H_4a_ and H_4b_, provided evidence about the electron density changes of these aromatic rings (Figure 1). Their host–guest complexation was further confirmed by ESI-MS (Figure S11). Two peaks attributed to the loss of one or two hexafluorophosphate counterions, [**4**⊃**6** – PF_6_]^+^ and [**4**⊃**6** – 2PF_6_]^2+^, were observed at *m*/*z* = 1134.4 and 494.7, respectively. Peaks related to other stoichiometries were not found.

**Figure 2:**
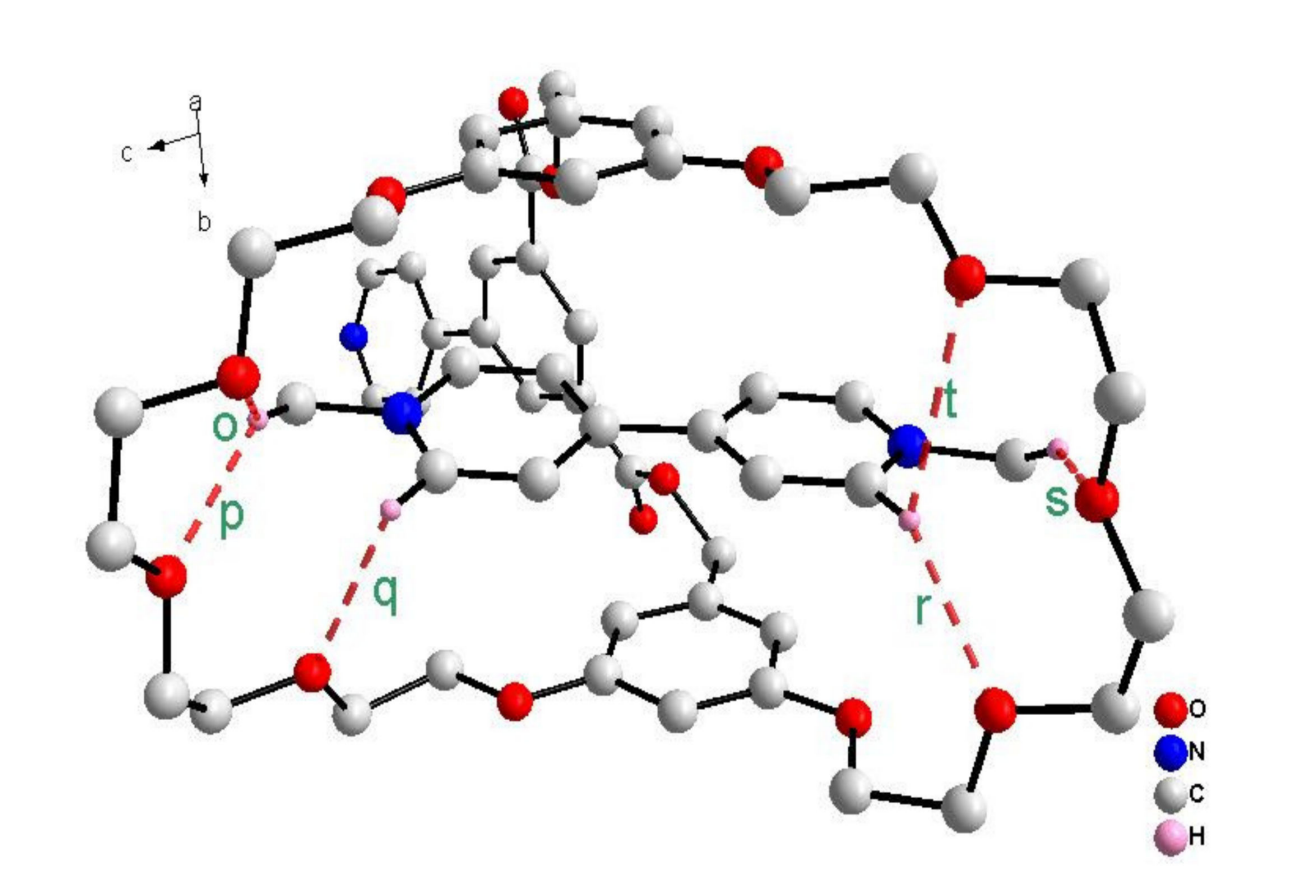
A ball-stick view of the X-ray structure of **4**⊃**6**. Counterions, solvent molecules, and hydrogens except the ones involved in hydrogen bonding are omitted for clarity. Hydrogen bond parameters: H^…^O (N) distance (Å), C-H^…^O (N) angle (deg) o, 2.536, 144.0; p, 2.475, 142.9; q, 2.803, 107.0; r, 2.431, 140.1; s, 2.469, 147.7; t, 3.012, 88.7.

To clarify the binding geometry of the complex, we tried to obtain suitable crystals of their host–guest complex for X-ray crystallographic analysis. Vapor diffusion of *n*-pentane into an acetone solution of the equivalent mixture of **4** and paraquat afforded **4**⊃**6** as yellow crystals. From the crystal structure, it was apparent that the third arm with the pyridyl group pointed to the outside of the cavity, making the host–guest recognition possible. Unlike the pseudorotaxanes formed by crown ethers and paraquat, **4**⊃**6** exhibits a taco complex geometry (Figure 2). In addition to the charge transfer interactions between the electron-rich aromatic rings of **4** and the electron-poor pyridinium rings of paraquat, CH^…^O hydrogen bonds played important roles during the complexation. From these results, we can confirm that, after functionalization with a pyridyl group on the third arm, cryptand **4** can still bind paraquat derivatives not only in solution but also in the solid state.

### 2.3. Preparation and Characterization of Di-Cryptand **8** and Tetra-Cryptand **9**

**Scheme 2:**
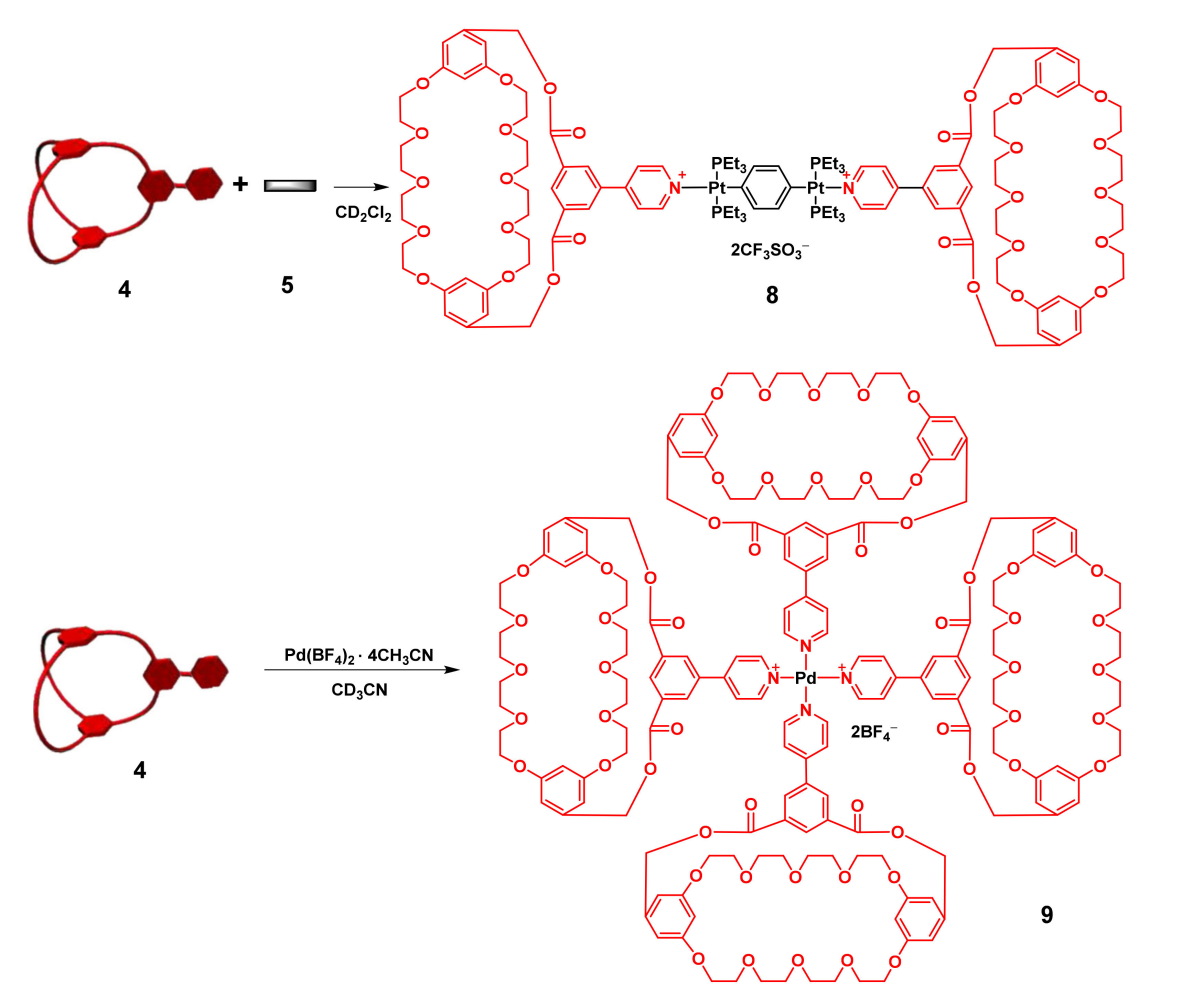
Preparation of the metalla-monomers with different topologies: di-cryptand **8** and tetra-cryptand **9**.

**Figure 3:**
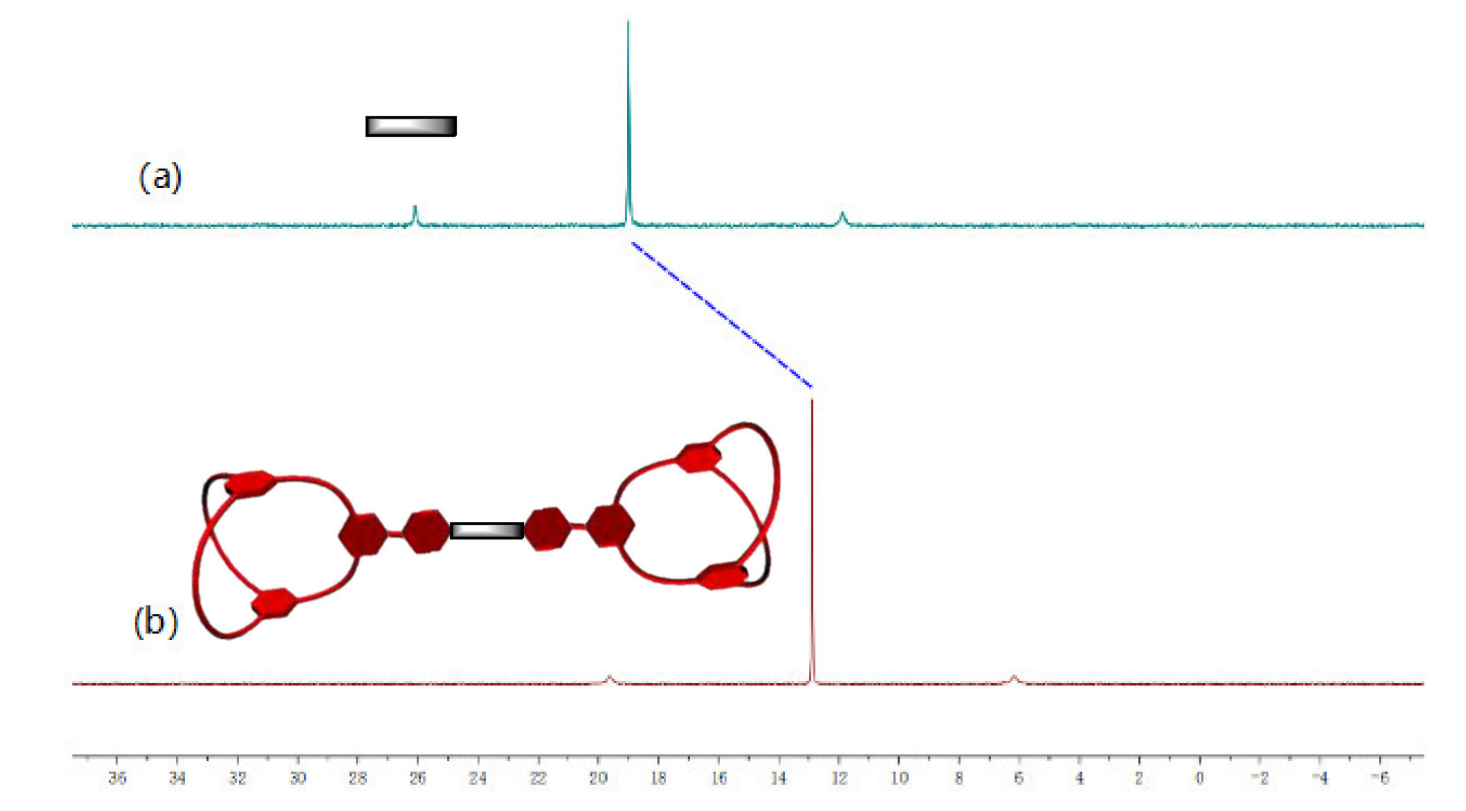
^31^P{^1^H} NMR spectra (202 MHz, CD_2_Cl_2_, 298 K): (**a**) the bidentate organoplatinum(II) acceptor **5** and (**b**) the di-cryptand monomer **8**.

**Figure 4:**
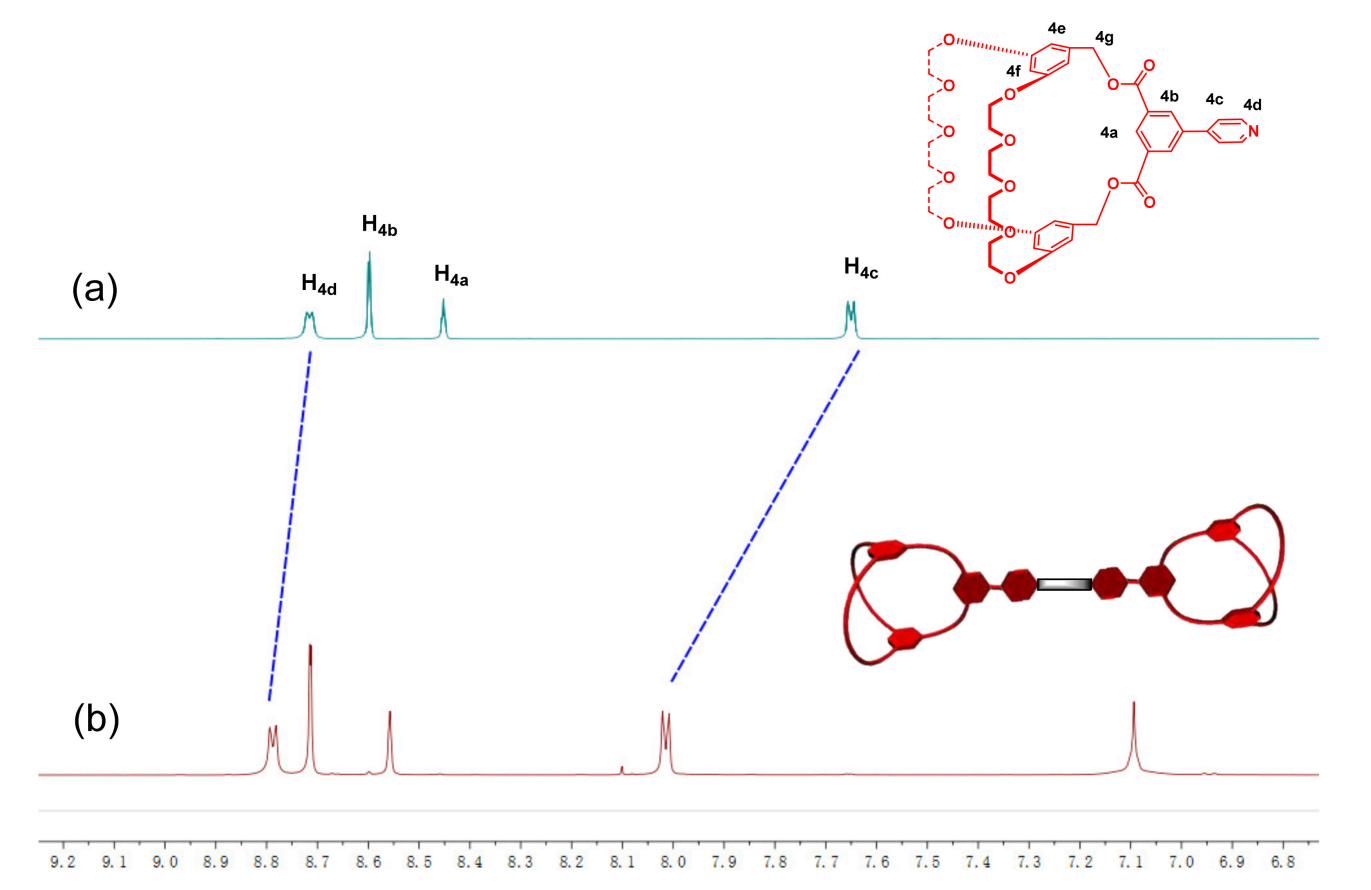
Partial ^1^H NMR spectra (500 MHz, CD_2_Cl_2_, 298 K): (**a**) the cryptand **4** and (**b**) the di-cryptand monomer **8**.

For the construction of supramolecular polymers with different topologies, two monomers (di-cryptand monomer **8** and tetra-cryptand monomer **9**) were prepared afterward, using **4** and different metal linkers, respectively (Scheme 2). Firstly, the linear di-cryptand monomer **8** was synthesized by mixing cryptand **4** (10.00 mM) and the bidentate organoplatinum(II) acceptor **5** (5.00 mM) in CD_2_Cl_2_ at room temperature. Multinuclear NMR (^1^H and ^31^P{^1^H}) analyses of the reaction solution indicated the formation of the desired discrete di-cryptand **8** (Figure S6 and Figure S7). The ^31^P{^1^H} NMR spectrum of **8** revealed a sharp singlet at 12.89 ppm with concomitant ^195^Pt satellites (*J*_Pt-P_ = 1359 Hz), indicating a single phosphorus environment (Figure 3b). The ^31^P peak of di-cryptand **8** shifted upfield by about 6.10 ppm compared with that of the bidentate organoplatinum(II) acceptor **5** (Figure 3). After coordination of the N atoms to the platinum centers of **5**, the protons of the pyridyl groups of **4** (especially H_4c_ and H_4d_) shifted downfield significantly in comparison with the free ones (Figure 4). Electrospray ionization time of flight mass spectrometry (ESI-TOF-MS) also provided convincing evidence for the formation of di-cryptand monomer **8**. A peak at *m*/*z* = 1272.9755 corresponding to [M – 2OTf]^2+^ was observed (Figure S13). It was isotopically resolved and agreed very well with the theoretical distributions.

Using the same method, a square monomer, tetra-cryptand **9**, was prepared by stirring a mixture of 4 equiv of cryptand **4** (10.00 mM) and 1 equiv of Pd(BF_4_)_2_•4CH_3_CN (2.50 mM) in CD_2_Cl_2_. Just like di-cryptand **8**, the ^1^H NMR spectrum showed very sharp peaks for each proton, which indicated the highly symmetric structure of tetra-cryptand **9** (Figure S8). Similar downfield chemical shifts (Figure S12) for signals of H_4c_ and H_4d_ were observed, indicating the formation of the metalla-monomer **9**. After storage in solution for 7 days, we recorded its ^1^H NMR spectrum again (Figure S12c). No significant changes could be found, which indicated that the coordination assembled tetra-cryptand **9** was stable at this condition. This is beneficial for the further preparation of cross-linked supramolecular polymers. The ESI-TOF-MS spectrum of **9** further confirmed its quadridentate structure. Two peaks at *m*/*z* = 1661.0897 for [M – 2BF_4_]^2+^ and 1119.7079 for [M – 2BF_4_ + K]^3+^ were observed (Figure S14). They were all isotopically resolved and agreed very well with the calculated distributions. Therefore, two metalla-monomers with different geometries (linear and square) for supramolecular orthogonal polymerization were successfully achieved through the coordination of cryptand **4** with corresponding metal linkers, respectively.

### 2.4. Formation and Characterization of Supramolecular Polymers

**Figure 5:**
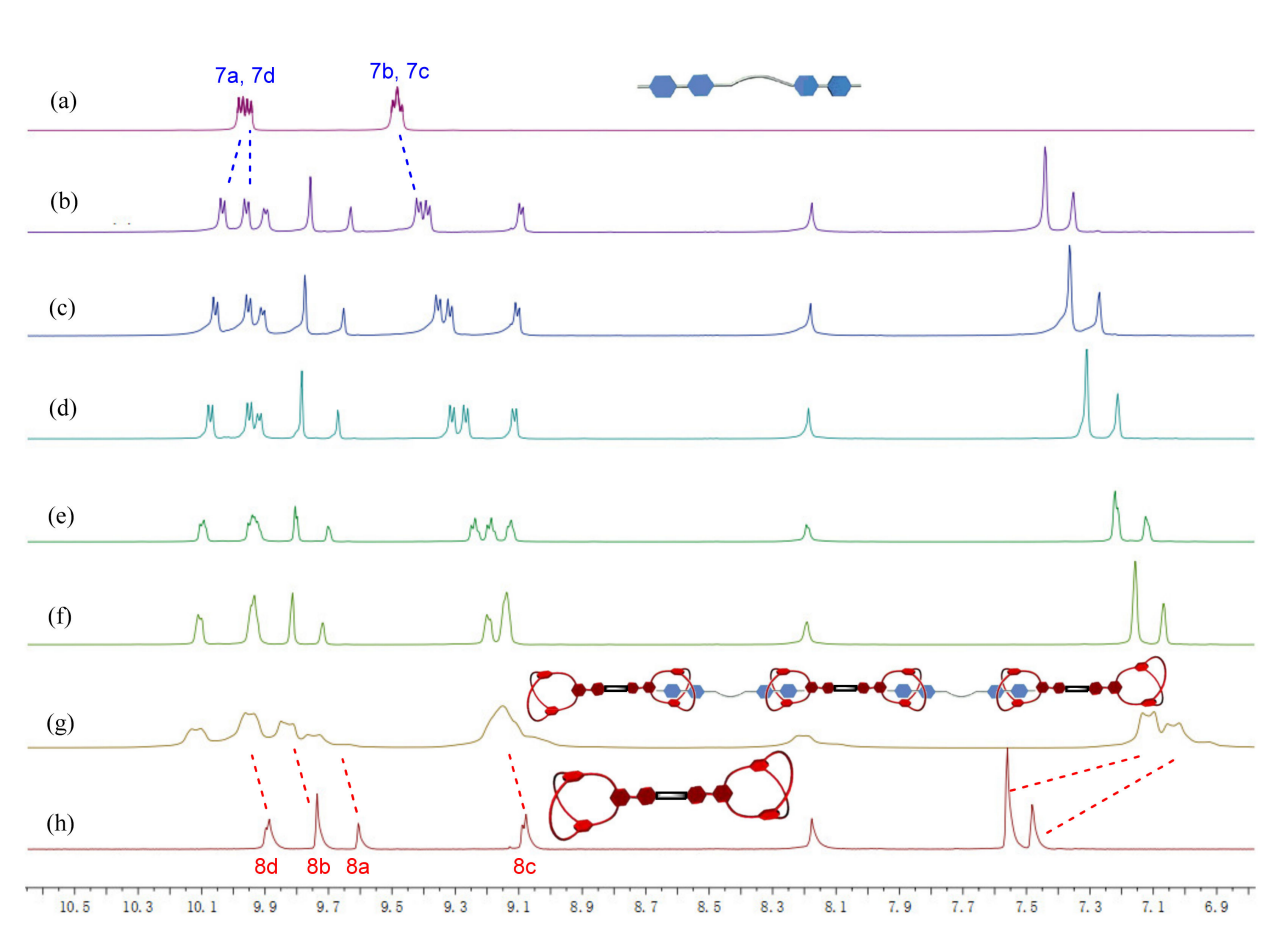
Partial ^1^H NMR spectra [500 MHz, CD_2_Cl_2_/CD_3_NO_2_ (1:1, *v/v*), 298 K]: (**a**) **7**; (b–g) solutions of **7** and **8** with 1:1 molar ratio at concentrations of (**b**) 2.50 mM, (**c**) 5.00 mM, (**d**) 10.0 mM, (**e**) 25.0 mM, (**f**) 50.0 mM, (**g**) 100.0 mM; (**h**) **8**. The assignments of protons were shown in Scheme 1.

Based on the two monomers, linear and cross-linked supramolecular polymers were constructed through the self-assembly of di-paraquat **7** with **8** and **9**, respectively. Because of the poor solubility of **7** in dichloromethane, all the following measurements were carried out in a mixed solvent of CD_2_Cl_2_/CD_3_NO_2_ (1:1, *v*/*v*). After equivalent di-cryptand **8** and di-paraquat guest **7** were mixed, the solution changed from colorless to deep-yellow immediately as a result of charge–transfer interactions, which showed authentic evidence for the strong host–guest complexation. We recorded the NMR spectra of the mixtures under a series of concentration gradients (Figure 5). They demonstrated that, just like the interactions between cryptand **4** and paraquat **6** mentioned above, the complexation of **8** and **7** is still in fast exchange on the ^1^H NMR timescale at room temperature. By comparing Figure 5a,b, it was found that peaks of H_7a_ and H_7d_ shifted downfield, whereas H_7b_ and H_7c_ shifted upfield, suggesting that the paraquat groups at the two peaks of **7** were deeply threaded into the cryptand cavity, like in the crystal structure of **4**⊃**6**. With the increase in concentrations, the distance between the two peaks of H_7a_ and H_7d_ gradually expanded, and H_7a_ shifted downfield significantly. Conversely, the two peaks of H_7b_ and H_7c_ shifted upfield and gradually converged into one broad peak. At the same time, all peaks of **7** and **8** turned from sharp into broad ones (especially at 100 mM, see Figure 5g). From the reported fast-exchanging host–guest systems, it can be inferred that the concentration-dependent proton NMR spectra of equivalent **7** and **8** reflect the gradual transformation of cyclic oligomers (at low concentrations) into linear supramolecular polymers (at high concentrations).

**Figure 6:**
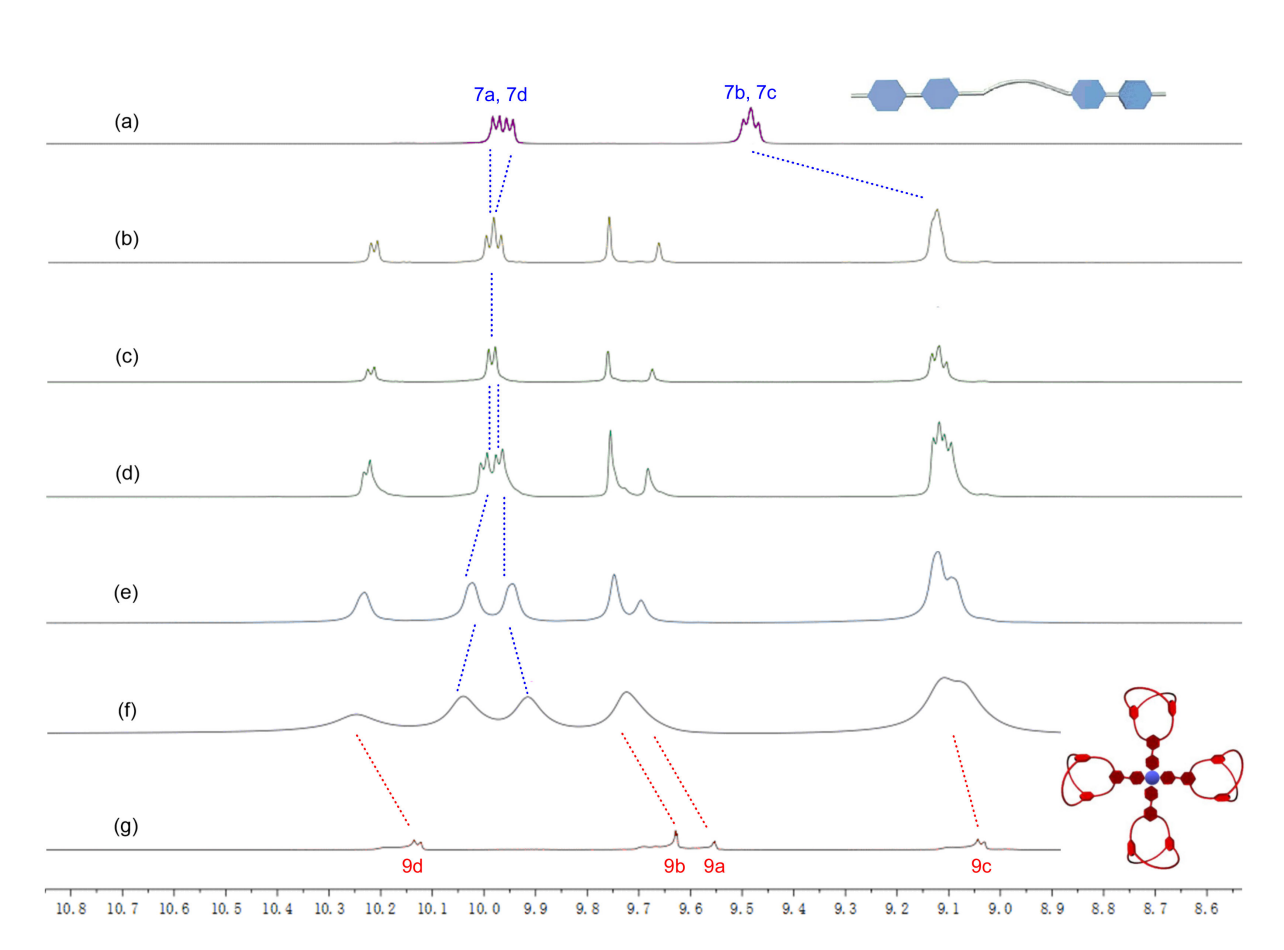
Partial ^1^H NMR spectra [500 MHz, CD_2_Cl_2_/CD_3_NO_2_ (1:1, *v*/*v*), 298 K]: (**a**) **7**; (b–g) solutions of **7** and **9** with 2:1 molar ratio at concentrations of (**b**) 2.50 mM, (**c**) 5.00 mM, (**d**) 10.0 mM, (**e**) 25.0 mM, (**f**) 100.0 mM; (**g**) **9**. The assignments of protons were shown in Scheme 1.

Similarly, a cross-linked supramolecular polymer was prepared by mixing 1 equiv of tetra-cryptand **9** with 2 equiv of di-paraquat **7** in the same solvent composed of CD_2_Cl_2_/CD_3_NO_2_ (1:1, *v/v*). The deep-yellow color of their mixture visually indicated charge transfer interactions. Then, we investigated their ^1^H NMR spectra (Figure 6) at different concentrations. When the concentration of the mixture increased from 5 mM to 100 mM, signals from **7** and **9** all changed from sharp into broad peaks gradually. However, in contrast to the spectra of **7** and **8**, no clear split of any proton peaks could be found for **7** and **9** until a concentration of 25 mM (Figure 6e), which is much lower than the linear one (until 100 mM, see Figure 5g), in accordance with their DOSY results. Peaks of H_7a_ and H_7d_ converged into one peak at first (Figure 6a–c) and then split into two peaks at high concentrations (Figure 6d–f). These phenomena probably illustrate that the cross-linked supramolecular polymer based on **7** and **9** has a lower critical polymerization concentration than the linear one (CPC, indicating a ring-chain transition from the cyclic oligomers into highly ordered polymers). From the above discussion, we preliminarily confirmed that both the linear and cross-linked supramolecular polymers could be constructed by mixing metalla-monomers (**8** and **9**) with di-paraquat **7** based on orthogonal self-assembly.

**Figure 7:**
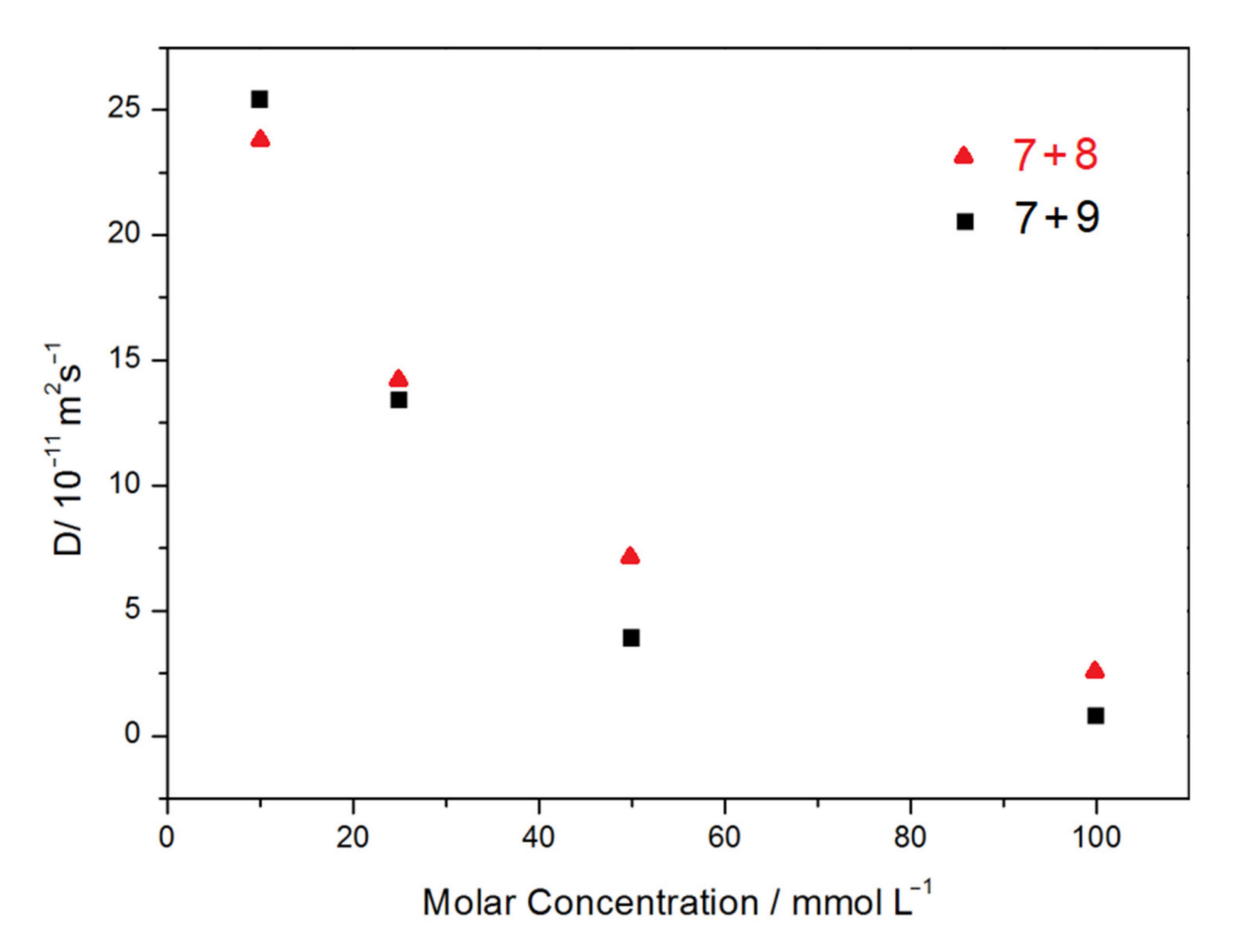
Concentration dependence of diffusion coefficient *D* [500 MHz, CD_2_Cl_2_/CD_3_NO_2_ (1 1, *v*/*v*), 298 K] of the linear (**7** + **8**) and the cross-linked (**7** + **9**) supramolecular polymer.

Two-dimensional diffusion-ordered ^1^H NMR spectroscopy (DOSY) experiments were performed to investigate the polymerization of **7** + **8** and **7** + **9**. Both of the aggregates are in fast exchange on the DOSY timescale. For the linear supramolecular polymer, as the concentrations of monomers (**7** and **8**) increased from 10 to 100 mM, the measured weight-average diffusion coefficients decreased from 2.34 × 10^–10^ to 0.22 × 10^–10^ m^2^s^–1^ (Figure 7, red), in accordance with the concentration dependence of their ^1^H NMR. Based on previous reports [21,40], it is well-known that a high degree of polymerization for the repeating unit is necessary to observe a sharp decrease in the diffusion coefficient. For the cross-linked system, when the concentration increased gradually, the diffusion coefficients decreased more significantly from 2.5 × 10^–10^ to 0.01 × 10^–10^ m^2^s^–1^ (Figure 7, black). When the concentrations were low, the diffusion coefficients of the linear and cross-linked systems were similar. However, when the concentrations were higher (>50 mM), the gap between their diffusion coefficients became larger. For instance, when both of their concentrations were 100 mM, the diffusion coefficient of the linear polymer was about 20 times larger than the cross-linked one, which proves the different average sizes of their aggregates. These results clearly indicate that the supramolecular polymerization process can be controlled not only by the binding constant of the host–guest system but also significantly by the topology of the monomer.

## 3. Materials and Methods

BMP32C10 diol [64], **5** [65], **6** [66], and **7** [67] were synthesized according to literature procedures. Pyridine-4-ylboronic acid, tetrakis(acetonitrile)palladium(II) tetrafluoroborate, and tetrakis(triphenylphosphine)palladium(0) were reagent grade and used as received. Dichloromethane was dried according to the typical procedures described in the literature [65]. The other solvents were employed as purchased. NMR spectra were collected on a Bruker AVANCE Ⅲ 500 spectrometer (Billerica, Massachusetts, USA) with the residual solvent or tetramethylsilane (TMS) as the internal standard. Crystal data were collected on a Bruker D8 Venture (Billerica, Massachusetts, USA). High-resolution electrospray ionization (HRESI) mass spectra were obtained on an Agilent 1290-6530 UPLC-Q-TOF spectrometer (Santa Clara, California, USA) equipped with an electrospray source. Melting points were measured on a Buchi M-565 electrothermal melting point apparatus (Flawil, Switzerland).

### 3.1. Synthesis of **1**

Under the protection of nitrogen, dimethyl 4-iodoisophthalate (1.0 g, 3.12 mmol), pyridine-4-ylboronic acid (576 mg, 4.69 mmol), K_3_PO_4_ (862 mg, 4.06 mmol), Pd(PPh_3_)_4_ (180 mg, 0.16 mmol), and 50 mL dimethyl formamide were added into a 100 mL three-necked flask. The mixture was stirred at 80 °C for 2 days, and it was then cooled to room temperature. After suction filtration, the solid was washed with dichloromethane (200 mL). The organic layer was dried over anhydrous Na_2_SO_4_ and evaporated to afford the crude product, which was isolated by column chromatography using petroleum ether/ethyl acetate (5:1, *v*/*v*) as the eluent. The fractions containing the product were combined and concentrated under vacuum to give **1** (651 mg, 70.6%) as a white solid. The ^1^H NMR spectrum of **1** is shown in Figure S1. ^1^H NMR (500 MHz, CDCl_3_, 298 K) *δ* (ppm): 8.75–8.73 (m, 3H), 8.50 (d, *J* = 1.3 Hz, 2H), 7.59 (d, *J* = 5.4 Hz, 2H), 3.99 (s, 6H).

### 3.2. Synthesis of **2**

Dimethyl 5-(pyridin-4-yl)isophthalate **1** (654 mg, 2.20 mmol) and sodium hydroxide (887 mg, 22.0 mmol) were dissolved in 50 mL of methanol. The mixture was stirred for 4 h under reflux. After the solvent was removed by rotary evaporation, distilled water (50 mL) was added. Dilute hydrochloric acid (1 M) was added dropwise until the pH of the solution was about 2–3. A white solid **2** (504 mg, 93.9%) precipitated from the aqueous phase and was collected by suction filtration. The ^1^H NMR spectrum of **2** is shown in Figure S2. ^1^H NMR (500 MHz, dimethyl sulfoxide-*d*_6_, 298 K) *δ* (ppm): 13.60 (br, 2H), 8.69 (d, *J* = 3.0 Hz, 2H), 8.51 (s, 1H), 8.33 (d, *J* = 1.2 Hz, 2H), 7.63 (d, *J* = 5.5 Hz, 2H).

### 3.3. Synthesis of **3**

5-(Pyridin-4-yl)isophthalic acid **2** (0.715 g, 2.94 mmol) and SOCl_2_ (10 mL) were refluxed under N_2_ for 12 h. After the excess SOCl_2_ was removed by distillation, the obtained white solid **3** was directly used in the next reaction.

### 3.4. Synthesis of **4**

BMP32C10 diol (1.25 g, 2.1 mmol) and freshly prepared 5-(pyridin-4-yl)isophthaloyl dichloride **3** (824 mg, 2.94 mmol) were dissolved in 50.0 mL freshly dried dichloromethane, respectively. The two solutions were loaded into plastic syringes whose metal needles were replaced with HPLC tubing and added via a syringe pump at 2.00 mL/h to a solution of pyridine (1.5 mL) and 4-dimethylaminopyridine (77 mg, 0.63 mmol) in freshly dried dichloromethane (500 mL). After the injection was complete, the reaction mixture was stirred for a further 3 days at room temperature. The solution was then washed with water (2 × 50 mL), dried over anhydrous Na_2_SO_4_, and evaporated to afford the crude product, which was isolated by column chromatography using CH_2_Cl_2_/CH_3_OH (100:1, *v/v*) as the eluent. The fractions containing the product were combined and concentrated under vacuum to give **4** as a white solid (434 mg, 45%): Mp 124.0‒125.3 °C. The ^1^H NMR spectrum of **4** is shown in Figure S3. ^1^H NMR (500 MHz, CDCl_3_, 298 K) *δ* (ppm): 8.70 (s, 2H), 8.56 (s, 3H), 7.64 (s, 2H), 6.49 (s, 4H), 6.46 (s, 2H), 5.23 (s, 4H), 4.00 (s, 8H), 3.79 (s, 8H), 3.68–3.65 (m, 16H). The ^13^C NMR spectrum of **4** is shown in Figure S4. ^13^C NMR (126 MHz, CDCl_3_, 298 K) *δ* (ppm):165.13, 160.10, 138.64, 137.11, 132.85, 131.55, 130.58, 122.34, 107.16, 101.51, 70.91, 70.71, 69.62, 67.67. ESI-TOF-MS for **4** is shown in Figure S5: HRESI-MS (*m*/*z*): Calcd. For [M + H]^+^: 804.3226; Found: 804.3213, error: –1.6 ppm. 

### 3.5. Synthesis of **8**

Cryptand **4** (8.04 mg, 10.0 mM) and **5** (6.18 mg, 5.00 mM) were mixed in CD_2_Cl_2_ at room temperature for 30 min to give the monomer **8**. The ^1^H NMR spectrum of **8** is shown in Figure S6. ^1^H NMR (500 MHz, CD_2_Cl_2_, 298 K) *δ* (ppm): 8.79 (d, *J* = 6.3 Hz, 4H), 8.71 (d, *J* = 1.4 Hz, 4H), 8.56 (t, *J* = 1.4 Hz, 2H), 8.02 (d, *J* = 6.6 Hz, 4H), 7.10 (s, 4H), 6.53 (d, *J* = 2.2 Hz, 8H), 6.44 (t, *J* = 2.1 Hz, 4H), 5.31 (s, 8H), 4.03 (dd, *J* = 5.1, 3.2 Hz, 16H), 3.76 (dd, *J* = 5.1, 3.2 Hz, 16H), 3.64–3.58 (m, 32H), 1.39–1.36 (m, 24H), 1.18–1.12 (m, 36H). The ^31^P{^1^H} NMR spectrum of **8** is shown in Figure S7. ^31^P{^1^H} NMR (202 MHz, CD_2_Cl_2_, 298 K) *δ* (ppm): 12.89 (s, ^195^Pt satellites, *J*_Pt-P_ = 2717 Hz). HRESI-TOF-MS: Calcd. For [**8** – 2OTf]^2+^ 1272.9834; Found: 1272.9755, error: –6.2 ppm.

### 3.6. Synthesis of **9**

Cryptand **4** (16.08 mg, 0.02 mmol) and Pd(BF_4_)_2_•4CH_3_CN (2.22 mg, 0.005 mmol) were mixed in CD_3_CN at room temperature for 2 h to give monomer **9**. The ^1^H NMR spectrum of **9** is shown in Figure S8. ^1^H NMR (500 MHz, CD_3_CN, 298 K) *δ* (ppm): 8.88 (d, *J* = 6.7 Hz, 8H), 8.50 (s, 8H), 8.32 (s, 4H), 7.91 (d, *J* = 6.8 Hz, 8H), 6.45 (d, *J* = 2.2 Hz, 16H), 6.35 (t, *J* = 2.1 Hz, 8H), 5.13 (s, 16H), 3.91 (dd, *J* = 5.1, 2.9 Hz, 32H), 3.63 (dd, *J* = 5.0, 3.1 Hz, 32H), 3.50–3.46 (m, 64H). HRESI-TOF-MS: Calcd. For [M − 2BF_4_]^2+^: 1661.0906; Found: 1661.0897, error: –0.5 ppm; Calcd. For [M − 2BF_4_ + K]^3+^: 1119.7079; Found: 1119.7065, error: –1.3 ppm.

### 3.7. X-ray Crystal Data for **4**⊃**6**

Crystallographic data: block, yellow, 0.38 × 0.30 × 0.16 mm^3^, C_58_H_69_F_12_N_3_O_15_P_2_, *FW* 1338.10, monoclinic, space group *P 2*1/n, *a* = 22.2283(4) Å, *b* = 10.6062(2) Å, *c* = 28.1146(6) Å, *α* = 90.00°, *β* = 111.381(1), *γ* = 90.00°, *V* = 6172.1(2) Å^3^, *Z* = 4, *D*_c_ = 1.440 g cm^−3^, *T* = 170 K, *µ* = 0.175 mm^−1^, 12593 measured reflections, 9619 independent reflections, 815 parameters, 0 restraint, *F*(000) = 2784, *R*_1_ = 0.0867, *wR*_2_ =0.1898, (all data), *R*_1_ = 0.0671, *wR*_2_ = 0.1722 [*I* > 2σ(*I*)], goodness-of-fit (*F*^2^) = 1.040. Deposition Number 2031213.

## 4. Conclusions

In summary, we have designed and prepared a novel cryptand **4** derived from BMP32C10 with a pyridyl group on the third arm pointing outside of its cavity. The host–guest recognition of **4** and paraquat was investigated by UV–vis, NMR, and ESI-MS spectroscopy. X-ray crystallographic analysis further proved the taco geometry of their 1:1 complex. Two metalla-monomers with different geometries were prepared subsequently through the coordination of **4** with the bidentate organoplatinum(II) acceptor **5** and the quadridentate Pd(BF_4_)_2_•4CH_3_CN, respectively. The linear di-cryptand **8** and the square tetra-cryptand **9** were characterized via multinuclear NMR spectra (^1^H and ^31^P{^1^H}) as well as ESI-TOF-MS. Furthermore, linear and cross-linked supramolecular polymers were self-assembled through the host–guest interactions of **8** and **9** with di-paraquat **7**, respectively. By comparing their ^1^H NMR and DOSY spectra at different concentrations, it was demonstrated that the supramolecular polymerization in solution was decided not only by the concentration of monomers and the strength of the host–guest recognition but also significantly by the topologies of the monomers. Considering the easy availability of coordination-driven metalla-monomers, the present study provides a new and simple way to fabricate supramolecular polymeric materials with controllable topology.

## Data Availability

The data presented in this study are available in insert article or supplementary material here.

## Supplementary Materials

The following are available online: Figure S1: 1H NMR spectrum (500 MHz, CDCl3, 298 K) of 1, Figure S2: 1H NMR spectrum (500 MHz, DMSO-d6, 298 K) of 2, Figure S3: 1H NMR spectrum (500 MHz, CDCl3, 298 K) of 4, Figure S4: 13C NMR spectrum (126 MHz, CDCl3, 298 K) of 4, Figure S5: ESI-MS spectrum of 4, Figure S6: 1H NMR spectrum (500 MHz, CD2Cl2, 298 K) of 8, Figure S7: 31P{1H} NMR spectrum (202 MHz, CD2Cl2, 298 K) of 8, Figure S8: 1H NMR spectrum (500 MHz, CD3CN, 298 K) of 9, Figure S9: UV–vis absorption spectrum of 2.00 mM 4 and 6 in acetone, Figure S10: Job plot showing the 1:1 stoichiometry of the complex of 4 and 6 in acetone, Figure S11: The positive electrospray ionization mass spectrum of an equimolar mixture of 4 and 6 in CH3CN, Figure S12: 1H NMR spectra (500 MHz, CD3CN, 298 K) of (a) cryptand 4; (b) tetra-cryptand 9; and (c) tetra-cryptand 9 after 7 days, Figure S13: Experimental (red) and calculated (blue) ESI-TOF-MS spectra of 8 [M – 2OTf]2+, Figure S14: Experimental (red) and calculated (blue) ESI-TOF-MS spectra of 9: (a) [M − 2BF4]2+ and (b) [M − 2BF4 + K]3+.

## Sample Availability

Samples of the compounds **4**, **5**, **6** and **7** are available from the authors.
