# Supplementary material for: Construction of Supramolecular Polymers with Different Topologies by Orthogonal Self-Assembly of Cryptand–Paraquat Recognition and Metal Coordination
Source: Molecules. 2021 Feb 11;26(4):952. doi: 10.3390/molecules26040952 (PMC7916833; doi:10.3390/molecules26040952)
Supplement: Supplementary file 1 [file molecules-26-00952-s001.pdf]

Construction of Supramolecular Polymers with Different Topologies by Orthogonal Self-Assembly of Cryptand–Paraquat Recognition and Metal Coordination

Kai Wang[†], Yuan-Guang Shao[†], Feng-Zhi Yan, Zibin Zhang* and Shijun Li*

College of Material, Chemistry and Chemical Engineering, Hangzhou Normal University, Hangzhou 310036 (P. R. China)

[†] K. Wang and Y.-G. Shao contributed equally to this work.

Email address: zzhang@hznu.edu.cn, l_shijun@hznu.edu.cn

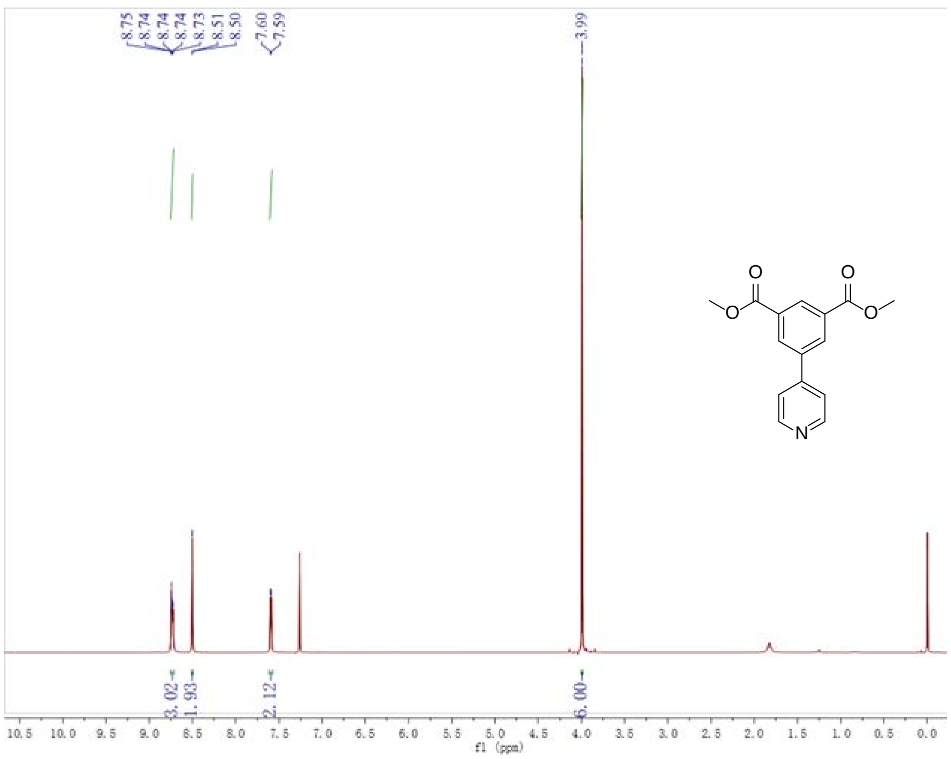

Figure S1. ^{1}H NMR spectrum (500 MHz, $CDCl_3$, 298 K) of **1**.

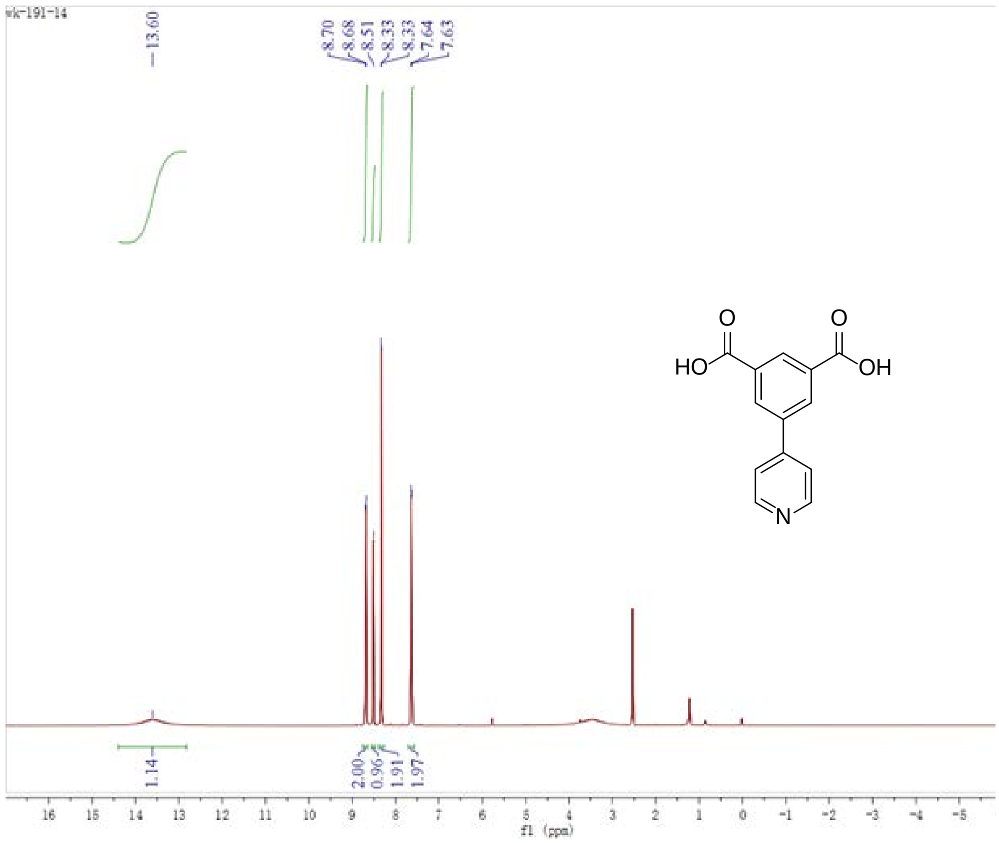

Figure S2. ^{1}H NMR spectrum (500 MHz, DMSO-d_6, 298 K) of **2**.

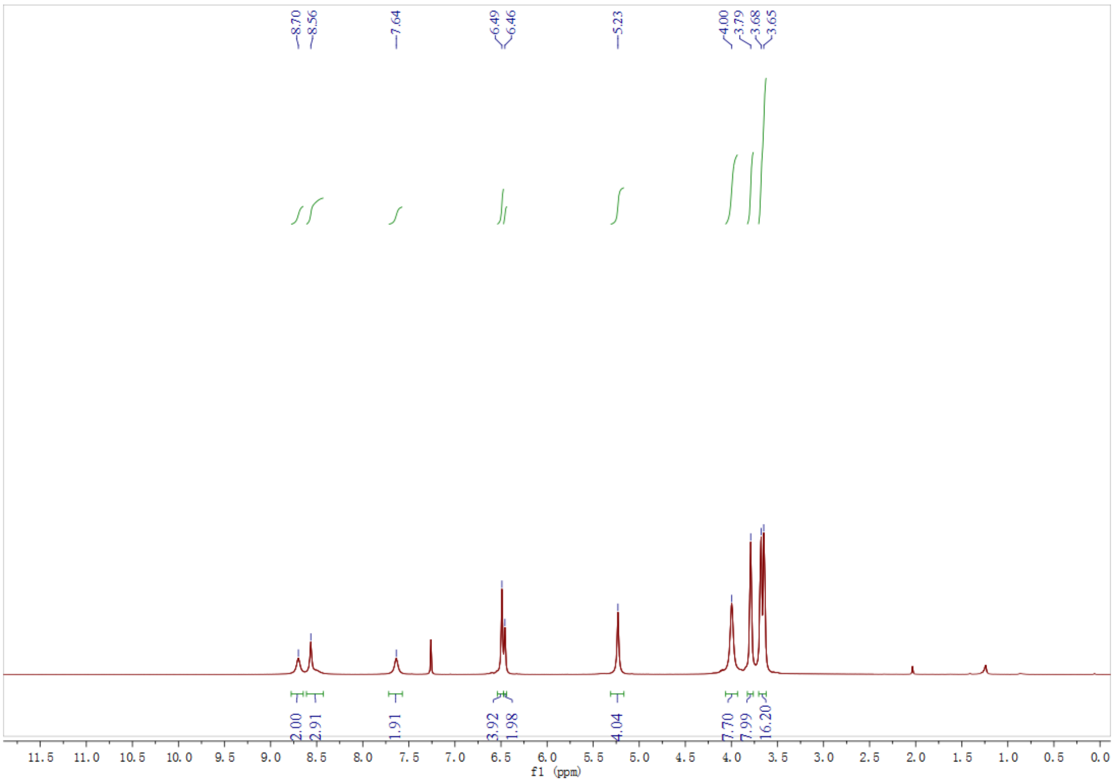

Figure S3. ^{1}H NMR spectrum (500 MHz, $CDCl_3$, 298 K) of **4**.

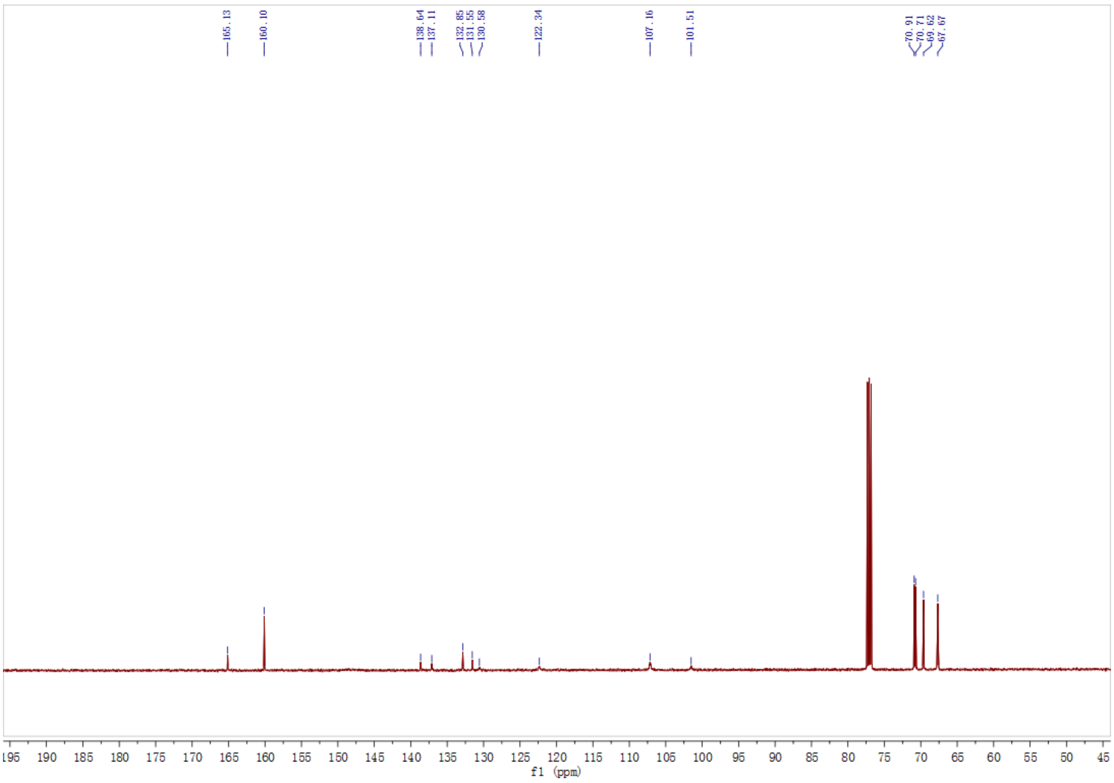

Figure S4. ^{13}C NMR spectrum (126 MHz, $CDCl_3$, 298 K) of **4**.

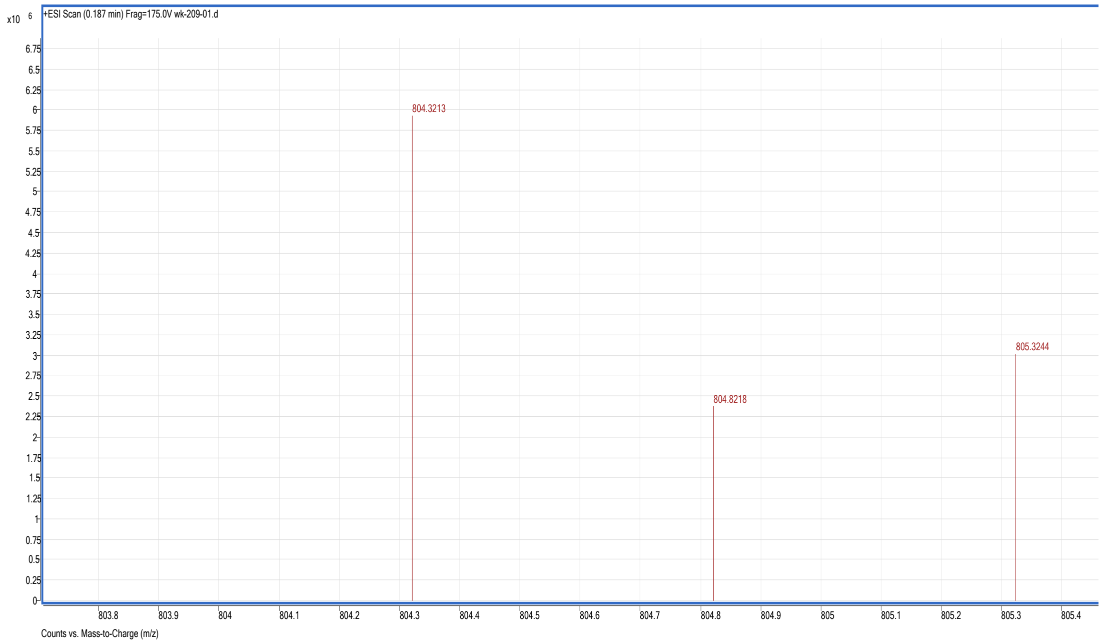

Figure S5. ESI-MS spectrum of **4** (m/z = 804.3213 $[M + H]^+$).

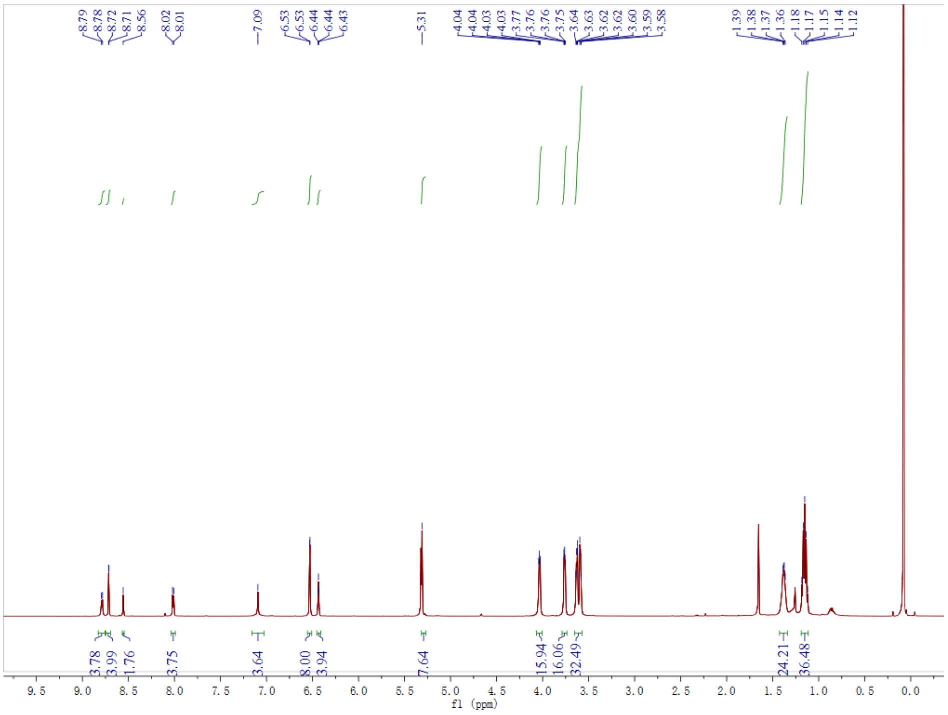

Figure S6. ${}^{1}H$ NMR spectrum (500 MHz, CD_2Cl_2, 298 K) of **8**.

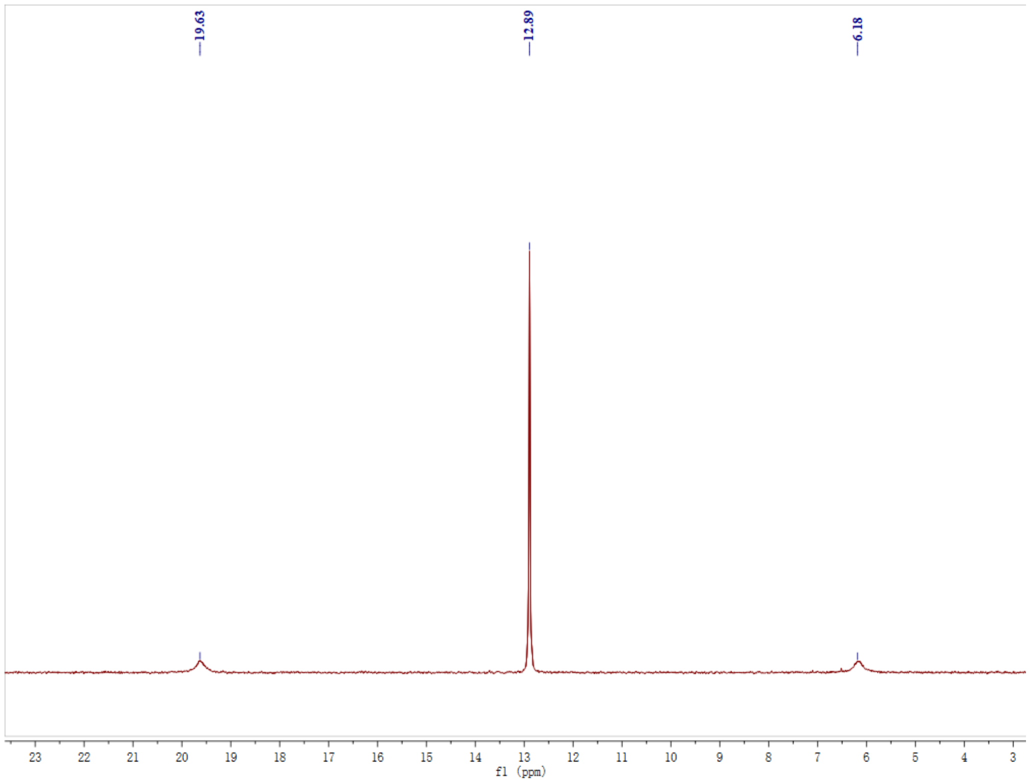

Figure S7. $^{31}P\{^1H\}$ NMR spectrum (202 MHz, CD_2Cl_2, 298 K) of **8**.

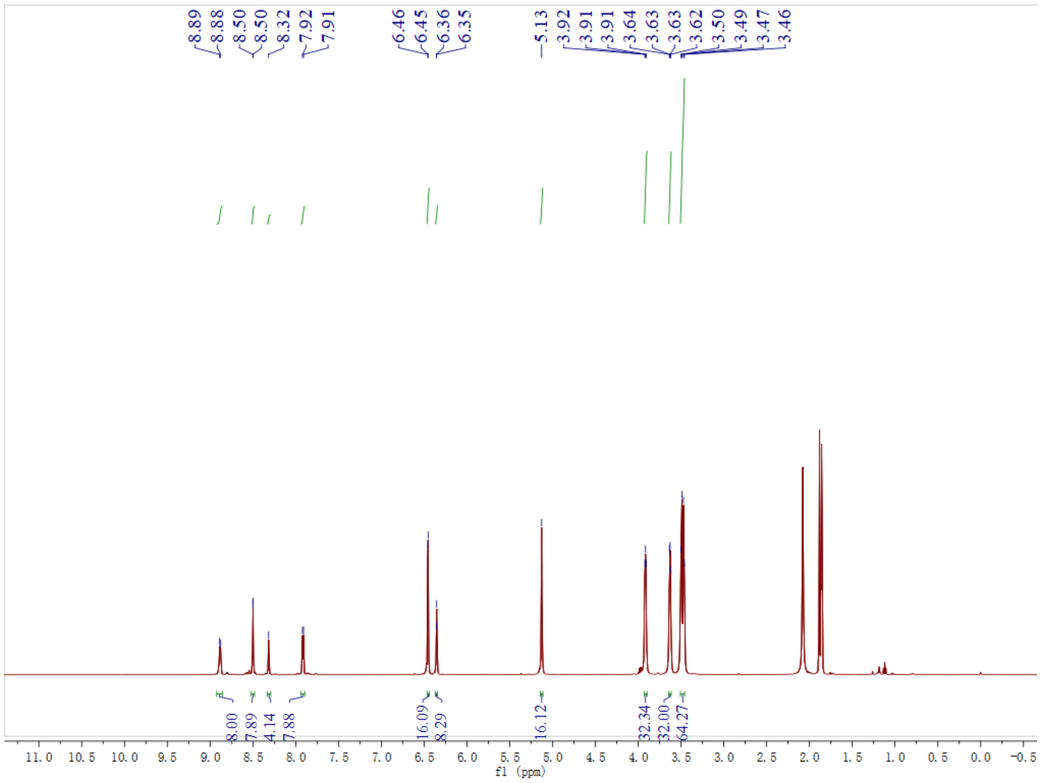

Figure S8. ^1H NMR spectrum (500 MHz, CD_3CN, 298 K) of **9**.

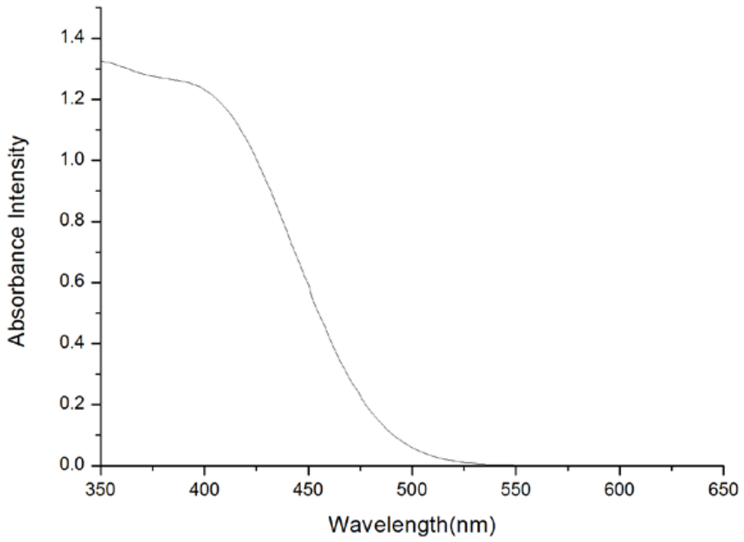

Figure S9. UV-vis absorption spectrum of 2.00 mM **4** and **6** in acetone.

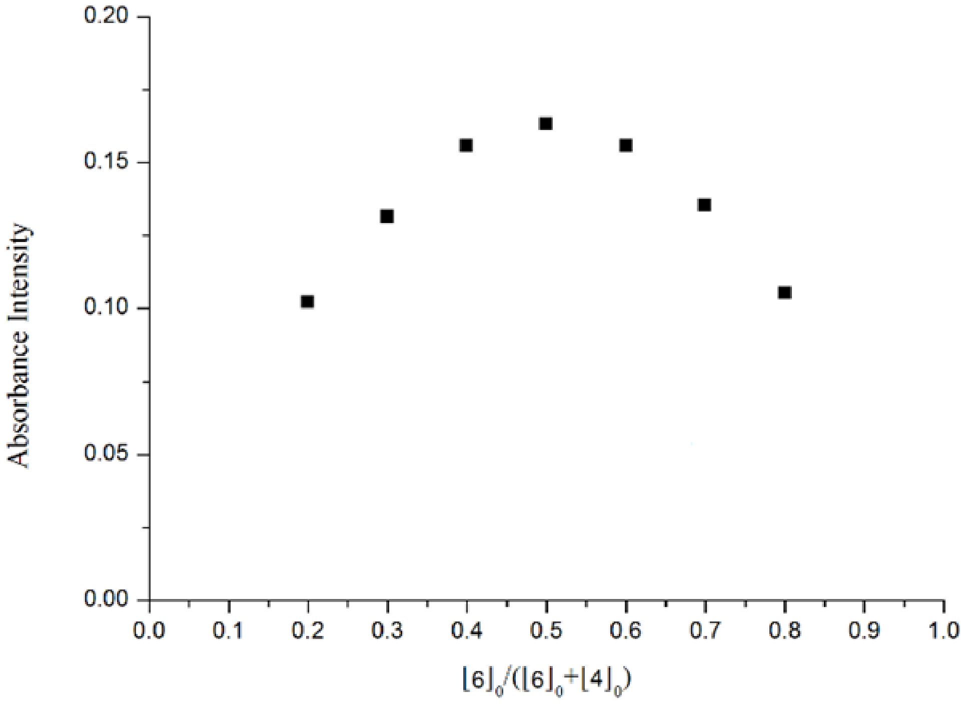

Figure S10. Job plot showing the 1:1 stoichiometry of the complex of **4** and **6** in acetone. $[\mathbf{4}]_0 + [\mathbf{6}]_0 = 2.00$ mM, $\lambda = 400$ nm; $[\mathbf{4}]_0$ and $[\mathbf{6}]_0$ are the initial concentrations of **4** and **6**.

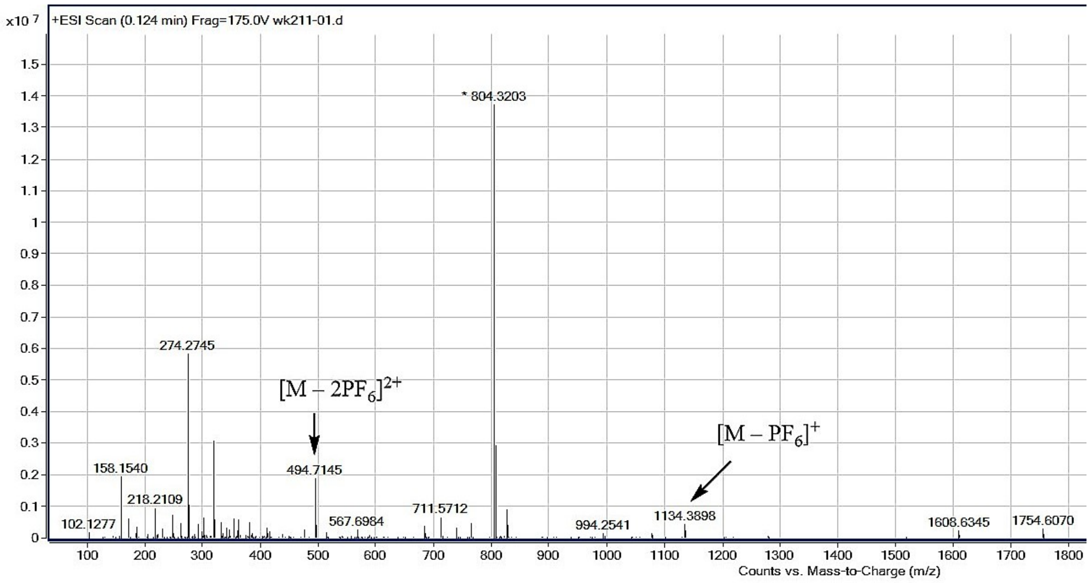

Figure S11. The positive electrospray ionization mass spectrum of an equimolar mixture of **4** and **6** in CH_3CN. Mass fragment at *m*/*z* 494.7145 for [**4**⊃**6** – $2PF_6]^{2+}$ and 1134.3898 for [**4**⊃**6** – $PF_6]^{+}$ confirmed the 1:1 complexation stoichiometry.

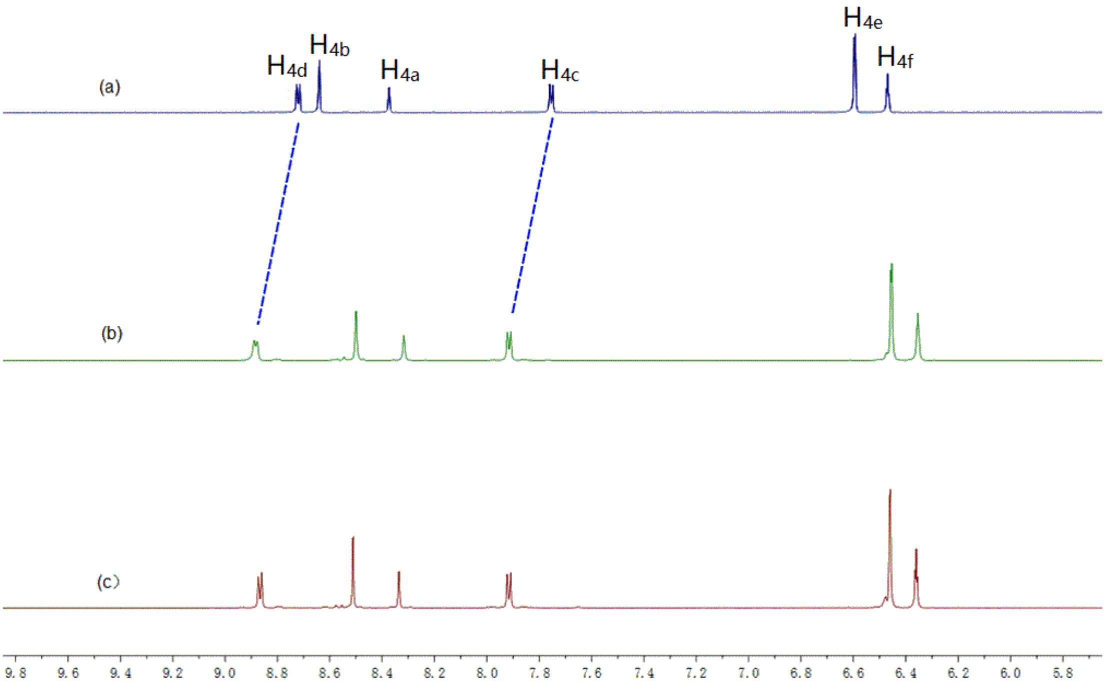

Figure S12. ^{1}H NMR spectra (500 MHz, CD_3CN, 298 K) of (a) cryptand **4**; (b) tetra-cryptand **9** and (c) tetra-cryptand **9** after 7 days.

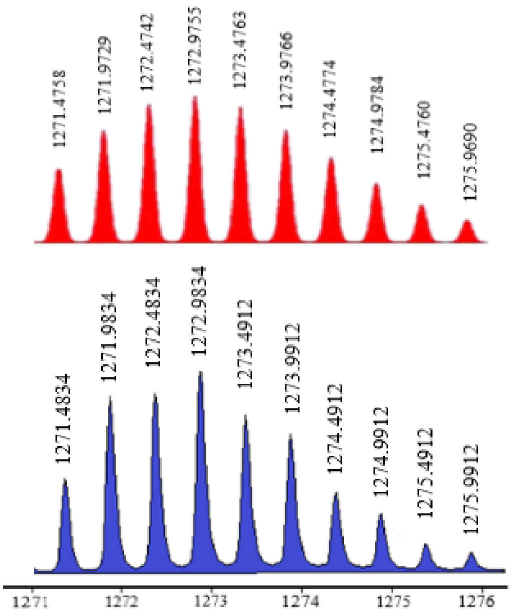

Figure S13. Experimental (red) and calculated (blue) ESI-TOF-MS spectra of **8** [M– 2OTf]$^{2+}$.

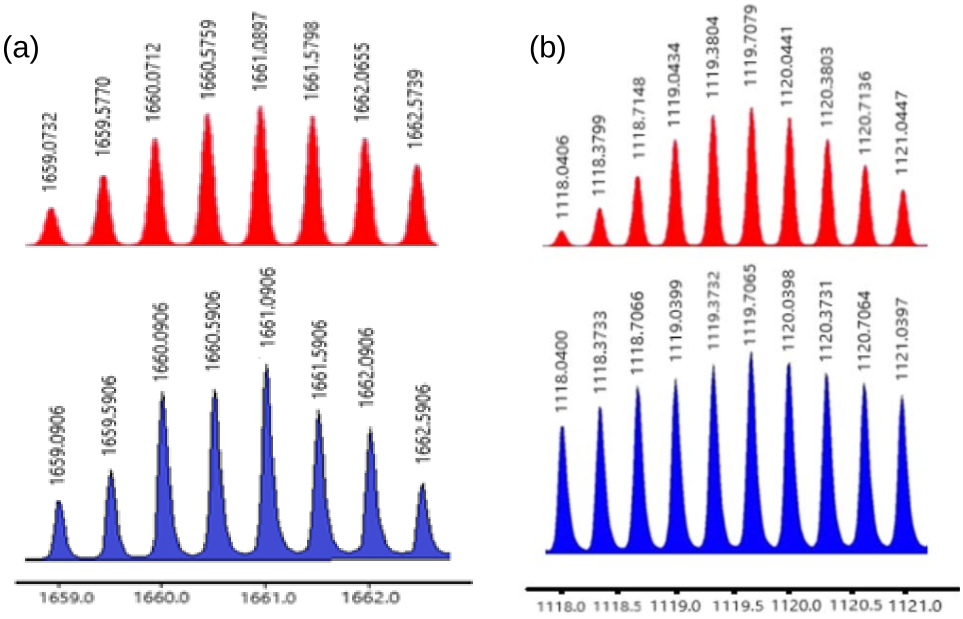

Figure S14. Experimental (red) and calculated (blue) ESI-TOF-MS spectra of **9**: (a) [M – $2BF_4]^{2+}$ and (b) [M – $2BF_4$ + K]$^{3+}$.
